# First Report of the Joint Exposure to Glyphosate and Glufosinate of a Male Population in the Province of Córdoba (Argentina)

**DOI:** 10.3390/toxics11121020

**Published:** 2023-12-14

**Authors:** Iohanna Filippi, Rocío I. Bonansea, Mariana Butinof, Ricardo A. Fernández, Marta Llorca, Marinella Farré, Sonia E. Muñoz, María V. Amé

**Affiliations:** 1 Instituto de Investigaciones en Ciencias de la Salud (INICSA), Consejo Nacional de Investigaciones Científicas y Técnicas (CONICET), Córdoba 5000, Argentina; ifilippi@unc.edu.ar (I.F.); ribqam@cid.csic.es (R.I.B.); smunoz@fcm.unc.edu.ar (S.E.M.); 2 Facultad de Ciencias Químicas, Universidad Nacional de Córdoba, Córdoba 5000, Argentina; 3 Escuela de Nutrición, Facultad de Ciencias Médicas, Universidad Nacional de Córdoba, Córdoba 5000, Argentina; mariana@butinof.com.ar; 4 Facultad de Ciencias de la Salud, Universidad Católica de Córdoba, Córdoba 5000, Argentina; ricardoantoniofernandez@yahoo.com.ar; 5 Department of Environmental Chemistry, Institute of Environmental Assessment and Water Research (IDÆA-CSIC), 08034 Barcelona, Spain; mlcqam@cid.csic.es (M.L.); mfuqam@cid.csic.es (M.F.); 6 Centro de Investigaciones en Bioquímica Clínica e Inmunología (CIBICI), Consejo Nacional de Investigaciones Científicas y Técnicas (CONICET), Córdoba 5000, Argentina

**Keywords:** glyphosate, glufosinate, environmental and occupational exposure, plasma, urine, UPLC-MS/MS

## Abstract

Despite potential health implications, data on the presence of Glyphosate (GLY) and other non-GLY herbicides in human matrices remain scarce. This study aimed to develop a simple and cost-effective methodology for detecting and quantifying GLY, its primary biodegradation product; aminomethylphosphonic acid (AMPA); and glufosinate (GLU) in plasma and urine of environmentally and occupationally exposed populations from the province of Córdoba (Argentina). Different alternatives of pre-treatment, derivatization with FMOC-Cl, solid phase extraction, and final sample conditioning steps were evaluated to improve the quantification of the herbicides by a high-performance liquid chromatography system coupled to a triple-quadrupole mass spectrometer. Recoveries ranged from 39 to 84% in both matrices, while limits of quantification were 3, 1, and 0.3 ng/mL and 3.6, 5.1, and 0.3 ng/mL for AMPA, GLY, and GLU in plasma and urine, respectively. In plasma samples, GLY was the most frequently detected analyte (32%), followed by GLU (10%). In urine samples, GLU was the most frequently detected herbicide (13%), followed by GLY (6%). No differences between group or matrix correlations were found. This study is the first report of GLU in human biological matrices and should be used to establish baseline values for future surveillance systems.

## 1. Introduction

Glyphosate (N-[phosphonomethyl]-glycine; GLY) and glufosinate (ammonium DL-homoalanin-4-(methyl) phosphinate; GLU) are broad-spectrum non-selective post-emergence herbicides used for weed and vegetation control. The herbicides GLY and GLU were introduced into the phytosanitary market in 1974 and 1981, respectively, and increasing sales volumes were reported worldwide over the years [1,2]. Indeed, their use has climbed since the development of genetically modified resistant crops in the 1990s [3]. The main product of biodegradation of GLY is aminomethylphosphonic acid (AMPA). Toxicity studies have shown that AMPA has comparable toxicological effects on its parent compound [4].

Given the extensive global utilization of these herbicides, various initiatives have been undertaken to assess their potential health impacts across diverse populations. Respiratory diseases, neurological effects, and congenital effects were positively associated with GLY exposure [5]. Moreover, regulatory agencies approach their toxicity based on literature evidence. Many of these agencies (e.g., the European Food Safety Agency (EFSA), the Food and Agricultural Organization of the United Nations (FAO-JMPR), and the United States Environmental Protection Agency (USEPA)) classify GLY as unlikely to pose a carcinogenic risk to humans. However, on the contrary, in 2015, the International Agency for Research on Cancer (IARC) posed GLY as probably carcinogenic to humans (Group 2A). Nevertheless, epidemiological studies did not provide the analysis of GLY in human matrices. Therefore, the safety and toxicity of these herbicides need to be deeply investigated.

Argentina is one of the world leaders in the production and export of agricultural products and is the second-largest country in crop production in Latin America [6]. In our country, since 1996, an extensive agricultural model based on transgenic soybean crops has been established. The model led to the widespread use of the herbicide GLY to control weeds in the crops of this oilseed, a strategy that later began to be extended to the cultivation of other species. Consequently, the volume of pesticide applications increased, with herbicides, mainly GLY, being the top-sold products in the phytosanitary market during the last decades. In recent years, a notable increase in the sale of herbicides has occurred and yet noGLY has been reported due to weed resistance [7]. As in the rest of the country, in the province of Córdoba, the frontiers of farming have expanded, and, in the 2019/2020 period, it was the main province of corn production, the second with the highest soybean production, and the third in wheat production of the country [8]. The herbicide GLY is used in sowing during and after the harvest of genetically modified soybean and corn crops. For wheat, GLY is used before the sowing and as a pre-harvest desiccant.

Concentrations of GLY and AMPA were reported in fresh water, sediment, and suspended particulate matter from different rivers in Argentina [9,10,11,12,13]. Glyphosate and AMPA were also detected in the local soil of productive systems [14,15] and, recently, in respirable dust (particulate matter finer than 10 μm in diameter) [16]. The presence of GLU, in addition to GLY and AMPA, was reported only in water samples of dairy farms from the province of Santa Fe (Argentina) [17].

Populations are exposed to environmental contaminants in different ways. The general population is mostly orally exposed through the consumption of contaminated food and drinks. Populations living near agricultural areas can also be exposed through inhalation of contaminated air, and, in addition, dermal contact is an important way of exposure in occupational settings [18]. With regard to human exposure, there is only one study reporting concentrations of GLY in the urine of a rural population from Chaco (Argentina) [19].

Physical–chemical characteristics of GLY, AMPA, and GLU (water solubility, high polarity, low molecular weight, and amphoteric property) make their detection particularly complex. The most used detection methods include chromatographic techniques, such as gas chromatography (GC), coupled with mass spectrometry (MS), tandem mass spectrometry (MS/MS), liquid chromatography (LC) coupled with fluorescence, ultraviolet, MS, or MS/MS [20]. Techniques like enzyme-linked immunosorbent assay, capillary electrophoresis, and nuclear magnetic resonance are also used to detect the compounds but these methods were less explored [21]. Independently of the detection methodology, different processes of sample preparation are needed to effectively detect these analytes. The more complex the matrix of the sample, the more pre-treatment will be necessary to clean up and concentrate the analytes before the detection. Solid phase extraction and liquid–liquid extraction (SPE and LLE, respectively) using a combination of organics and/or non-organic solvents are the most widely used strategies to clean up and concentrate the analytes of interest from complex matrix samples [10,17,22,23]. Another key issue to feasibly detect these highly polar and low-weight compounds is the derivatization step. Either pre- or post-column derivatization methods have been developed for LC detection. The 9-fluorenmethylcholoroformate (FMOC-Cl) is the most common pre-column derivatizer, while O-phthalaldehyde and 2-mercaptoethanol are the most common post-column derivatizers used [20]. Direct LC determination of the herbicides, avoiding derivatization steps, has also been purposed but generally with lower performance of the quality assurance parameters [24]. Regarding GC detection, acylation–esterification reactions are used to obtain less polar and more volatile derivatives [21]. Nevertheless, LC is the most selected method to determine these phosphonic and amino acid herbicides because of the possibility of using a reversed-phase column for chromatographic separation and the advantage of performing the derivatization steps in aqueous solutions [21].

The aim of this study is to develop a simple cost effective sensitive technique to detect and quantify GLY, GLU, and AMPA in plasma and urine of environmentally and occupationally exposed populations from the province of Córdoba (Argentina) and to compare results with available data of these herbicides exposure.

## 2. Materials and Methods

### 2.1. Chemicals and Reagents

Pesticidestandards of GLY, AMPA, and GLU were obtained from (Sigma-Aldrich, St. Louis, MO, USA). Individual pesticide stock standard solutions were prepared by dissolving pesticides in HPLC-grade water. Derivatizing agent, 9-fluorenmethylcholoroformate (FMOC-Cl), was obtained from Sigma-Aldrich (St. Louis, MO, USA). Tripotassium phosphate (K_3_PO_4_), ammonium acetate (C_2_H_7_NO_2_), disodium tetraboratedecahydrate (Na_2_B_4_O_7_), formic acid, chloride acid (HCl), and sodium hydroxide (NaOH) were obtained from J.T. Baker (Philipsburg, NJ, USA). Methanol (MeOH) and acetonitrile (ACN) of HPLC grade were obtained from J.T Baker (Philipsburg, NJ, USA). Solid phase extraction cartridges (OASIS HLB and WAX) were obtained from (Waters, Milford, MA, USA). Fetal bovine serum was obtained from Sigma-Aldrich (St. Louis, MO, USA).

### 2.2. Extraction Procedure

#### 2.2.1. Plasma

Pre-treatment of the samples consisted of obtaining protein-free plasma by adding 100 µL of ACN to 100 µL of plasma followed by vortexing and centrifugation at 20 °C (10 min-1699 g). After acidification with 0.3 M HCl to reach pH = 1, samples were agitated (1 h) and neutralized with NaOH 3 M to reach pH = 7 [25]. Derivatization of the samples was performed using FMOC-Cl [10,25]. First, the volume was adjusted to 0.8 mL with HPLC-grade water. Then, 0.1 mL of borate buffer (40 mM, pH = 9) and 0.1 mL of FMOC-Cl 6.5 mM (prepared by dissolving 168 mg of FMOC-Cl in 100 mL of ACN) were added. The reaction was allowed to take place during 2 h in darkness and agitation and stopped by adding formic acid 1% to reach pH = 3. Samples were centrifuged at 20 °C (1 min-1699 g) and the supernatant was transferred to a new polypropylene tube. In total, 5 mL of HPLC-grade water and 0.1 mL of EDTA 4% were added. Solid phase extraction (SPE) cartridges OASIS HLB (3 cm^3^, 200 mg) were used to extract the analytes. The conditioning of the cartridges was performed with 2 × 2.5 mL of MeOH (HPLC) and 2 × 2.5 mL of HPLC water (0.1% formic acid, pH = 3). Then, the samples were passed through the cartridges and the elution was performed with 2 × 2.5 mL of MeOH (HPLC). Finally, the extracts were concentrated under a gentle stream of nitrogen and reconstituted in 100 µL of MeOH:ammonium acetate 5 mM (50:50 *v*/*v*) and centrifuged (10 min-1699 g). The outline of the procedure is shown in Table S1.

#### 2.2.2. Urine

Different methodologies were compared to quantify the analytes in urine (Table S2).

Methodology A: The same methodology as that described on plasma samples was applied to urine samples. In this case, the deproteinization step was not necessary and the volumes of samples were adjusted. Briefly, 1 mL of centrifuged urine (10 min-1000 g) was acidified with HCl 0.3 M to reach pH = 1, agitated for 1 h, and neutralized with NaOH 3 M to reach pH = 7. For derivatization, the volume of samples was adjusted to 8 mL with HPLC-grade water. Then, 1 mL of borate buffer (40 mM, pH = 9) and 1 mL of FMOC-Cl 6.5 mM were added and samples were shaken for 2 h in darkness. Formic acid of 1% was added to reach pH = 3 and to stop the derivatization step. Samples were centrifuged at 20 °C (1 min-1699 g) and the supernatant was transferred to a new propylene tube. In total, 10 mL of HPLC-grade water and 0.4 mL of EDTA 4% were added. Solid phase extraction (SPE) cartridges OASIS HLB (3 cm^3^, 200 mg) were used to extract the analytes of interest. Conditioning of the SPE cartridges was performed with 2 × 2.5 mL of MeOH (HPLC) and 2 × 2.5 mL of HPLC water (0.1% formic acid, pH = 3). Then, samples were passed through the cartridges and the elution was performed with 2 × 5 mL of MeOH (HPLC). Finally, 1 mL of the extract was concentrated under a gentle stream of nitrogen and reconstituted in 100 µL of MeOH:ammonium acetate 5 mM (50:50 *v*/*v*) and centrifuged (10 min-1699 g).

Methodology B: To 1 mL of centrifuged urine, 0.4 mL of EDTA (4%, pH = 3) was added. Samples were agitated and let rest for 15 min. Acidification, neutralization, and derivatization steps were performed identically as in methodology A. In total, 10 mL of HPLC-grade water and 0.4 mL of EDTA 4% were added. Solid phase extraction was also performed identically as in methodology A.

Methodology C: One mL of NaOH 0.6 M was added to 1 mL of centrifuged urine [22]. Samples were agitated 1 h, and neutralized by adding HCl 3 M to reach pH = 7. Derivatization and SPE steps were performed identically as in methodology A.

Methodology D: Methodology A was reproduced, with higher concentration of the FMOC-Cl (12 mM) [22].

Methodology E: Methodology A was applied in this assay, but the derivatization step was modified with the objective of dilute the sample. In the derivatization step, the volume of the samples was adjusted to 16 mL with HPLC-grade water, 2 mL of borate buffer 40 mM and 2 mL of FMOC-Cl 6.5 mM were added. The following steps were performed identically as in methodology A.

Methodology F: In this assay methodology A was applied with a larger dilution of the sample. The volume of the samples was adjusted to 32 mL with HPLC-grade water, 4 mL of borate buffer 40 mM and 4 mL of FMOC-Cl 6.5 mM were added. The following steps were performed identically as in methodology A.

Methodology G: A further dilution of the samples in the derivatization step of methodology A was tested. The volume of the samples was adjusted to 80 mL with HPLC-grade water and 10 mL of 40 mM borate buffer and 10 mL of 6.5 mM FMOC-Cl were added. The following steps were performed identically as in methodology A.

Methodology H: Acidification, neutralization, and derivatization steps were performed identically as in methodology A but a different SPE cartridge, OASIS WAX (3 cm^3^, 60 mg), was used to extract the analytes of interest from samples. Conditioning of the SPE cartridges was performed with 2 × 2.5 mL of MeOH HPLC (0.1% NH_4_OH and 2 × 2.5 mL of HPLC water). Then, the samples (pH = 7) were passed through the cartridges and the elution was performed with 2 × 5 mL of MeOH HPLC (0.1% NH_4_OH). Finally, 1 mL of the extract was concentrated under a gentle stream of nitrogen and reconstituted in 100 µL of MeOH:ammonium acetate 5 mM (50:50 *v*/*v*) and centrifuged (10 min-1699 g).

Methodology I: Acidification and neutralization of the samples were performed as mentioned in methodology A. Derivatization steps were performed as mentioned in methodology F. Solid phase extraction was performed identically as in methodology H.

Methodology J: The acidification and neutralization of samples were performed as described in methodology A. Then, a previous extraction step was tested [10,14]. This step consisted of the addition of 9 mL of a solution containing Na_2_B_4_O_7_ and K_3_PO_4_, with 100 mM of each one. Steps of shaking for 1 min and sonication for 5 min were applied to the samples three times. Then, 1mL of FMOC-Cl 6.5 mM was added, samples were shaken for 2 h in darkness, and 1% formic acid was added to reach pH = 3 and to stop the derivatization step. Samples were centrifuged at 20 °C (1 min-1699 g) and the supernatant was transferred to a new propylene tube. Then, 10 mL of HPLC-grade water and 0.4 mL of EDTA 4% were added. Solid phase extraction was performed as described in methodology A.

Methodology K: Methodology J was reproduced but the extra extraction step was performed by adding 36 mL of the salt solution with the objective of diluting the sample. Consequently, for the derivatization step, 4 mL of the FMOC-Cl were added. Solid phase extraction was performed as described in methodology A.

### 2.3. Chromatographic Conditions

The identification and quantification of the pesticide metabolites were carried out using an ultra-performance liquid chromatography system Aria Mx with two Transcend quaternary pumps coupled to a TSQ Quantiva triple-quadrupole mass spectrometer equipped with a heated electrospray ionization (HESI) source (Thermo Fisher Scientific Inc., Waltham, MA, USA). Chromatographic separation was achieved on a Purospher^®^STAR RP-18 endcapped column, (150 × 2.1, 2 µm particle diameter) obtained from Merck (KGaA, Darmstadt, Germany). The column temperature was kept at 30 °C during the analysis. The injection volume was 10 μL at a flow rate of 0.3 mL/min. A gradient elution with a mobile phase of ammonium acetate (pH = 9) and MeOH was used for analysis. The gradient started with ammonium acetate:MeOH 90:10 to minute 3. From minute 3 to minute 10, the gradient was inverted to ammonium acetate:MeOH 10:90 and maintained for the next 7 min. From minute 17 to minute 19, the gradient was adjusted to the initial conditions and maintained for 1 min. The total run time was 20 min. Data MS/MS acquisition was performed in negative ionization mode. Table 1 shows the quantification (Q) and confirmation (C) of selected reaction monitoring (SRM) transitions for each compound. For identification purposes, each analyte should have fulfilled all of the following criteria: a-retention time (Rt) of the analyte in the sample should not be different in ±0.2 seg of the Rt of the analyte in the standard solution of the calibration curve; b-two m/z transitions were confirmed for every analyte; and c-the relative abundances of SRM (Q-SRM/C-SRM) in the sample should have been within ± 30% of the relative abundances obtained using standard solutions in the calibration curve.

Data were processed using the Thermo Xcalibur 3.0.63 software (Thermo Fisher Scientific Inc.).

### 2.4. Method Validation

Analytical quality parameters were measured using fetal bovine serum and the first morning urine sample of subjects with unknown exposure to pesticides for plasma and urine method validation, respectively. These matrices were spiked with known concentrations of the target compounds and treated identically as the samples. Blanks were frequently analyzed, with respect to every set of 10 to 15 samples, to ensure the absence of contaminants, carry over, or interferences arising from samples or laboratory handling. Calibration straight curves were prepared by adding different amounts of the pesticide standards to the different matrices to cover a wide range of concentrations (1 to 500 ng/mL). Limits of detection (LOD) and quantification (LOQ) were calculated considering the signal-to-noise ratio of 3 and 10, respectively. Precision and accuracy were evaluated at 10 ng/mL and obtained from three replicates. Precision was informed as the percentage of the relative standard deviation (% RSD) and accuracy as the percentage of the difference between the nominal and the calculated concentration. Percentages of recoveries (% Rec) were calculated at 10 ng/mL and checked for each different methodology. Concentration of the analytes in the real samples was achieved from the linear regression plot obtained for each standard analyte and for each batch of samples.

### 2.5. Study Area and Subjects

The region under study is one of the most important agricultural areas of the country. It is the region with the highest levels of production of soybeans and one of the three main producers of corn and wheat in the province [8]. Individuals with different scenarios of exposure were invited to participate of the study: occupationally exposed subjects (agricultural worker performing pesticides application tasks, such as loading, mixing, and/or spraying activities) and environmentally exposed subjects (individuals selected from the general population of the surrounding area who were not occupationally exposed to pesticides and who had similar socio-demographic conditions to the occupationally exposed population).

Those who agreed to participate signed a written informed consent before the sampling. Every subject involved in the study answered a survey to obtain socio-demographic data and provided biological samples. Sampling was carried out in September 2017, considered a period of high exposure for the occupationally exposed subjects. The research proposal was approved by the Ethics Review Committee of Hospital Nacional de Clínicas and is registered with the Ethics Committee of Health Investigations of the Province of Cordoba (RePIS N° 2732 y 044/10). The validation of exposure based on the use and application of pesticides was previously described for workers from the same agricultural geographic area and people from the surroundings [26].

Biological samples, blood, and urine were collected after an overnight fasting period and preserved cool until they reached the laboratory (4–8 °C). Plasma, from EDTA tubes, was separated by centrifugation (10 min-1500 g) and stored at −80 °C until pesticide analysis. First-morning urine samples were collected in sterile containers and stored at −80 °C until pesticide analysis.

### 2.6. Statistical Analysis

Socio-demographic characteristics of the population were described using a Student t-test and Chi2 test to compare continuous variables between groups of subjects and to observe possible associations between the exposure condition and categorical variables, respectively. Concentrations of the analytes were expressed in ng/mL and described as the geometric means (GM), confidence interval (CI), medians (M), and quantiles (Q). Detection frequencies (DF) were calculated using concentrations above the LOQ. The Mann–Whitney U test was used to compare the median concentration of the analytes between groups of subjects and the Spearman coefficient was used to observe correlations between the concentrations of the analytes in the different groups. The *p*-value was considered as significant when it was under 0.05. Statistical analyses were performed using Stata© v17.

## 3. Results

### 3.1. Method Optimization

#### 3.1.1. Urine

The different methodologies tested were compared by using the % Rec based on the non-addition of internal standards (IS) or isotope labeled internal standards (ILIS) to the samples (Table 2). According to these results, methodology K was selected for future urine sample analysis (Table 3). Briefly, 1 mL of centrifuged urine (10 min-1000 g) was acidified with HCl 0.3 M to reach pH = 1, agitated for 1 h, and neutralized with NaOH 3 M to reach pH = 7. Then, a previous extraction step was performed by adding 36 mL of a solution containing Na_2_B_4_O_7_ and K_3_PO_4_, with 100 mM of each one. Steps of shaking for 1 min and sonication for 5 min were applied to the sample three times. The derivatization step was performed with 4 mL of FMOC-Cl 6.5 mM and samples were shaken for 2 h in darkness and formic acid 1% was added to reach pH = 3 and to stop the derivatization step. Samples were centrifuged at 20 °C (1 min-1699 g), the supernatant was transferred to a new propylene tube, and 10 mL of HPLC-grade water and 0.4 mL of EDTA 4% were added. Conditioning of the SPE cartridges (OASIS HLB, 3 cm^3^, 200 mg) was performed with 2 × 2.5 mL of MeOH (HPLC) and 2 × 2.5 mL of HPLC water (0.1% formic acid, pH = 3). Then, samples were passed through the cartridges and the elution was performed with 2 × 5 mL of MeOH (HPLC). Finally, 1 mL of the extract was concentrated under a gentle stream of nitrogen and reconstituted in 100 µL of MeOH:ammonium acetate 5 mM (50:50 *v*/*v*) and centrifuged (10 min-1699 g).

#### 3.1.2. Plasma

The environmental methodology previously developed for the analysis of water samples [10,25] was adapted to the analysis of plasma samples. Results of the analytical quality parameters assessed for the purpose methodology showed good reliability of the method and no further trials were needed (Table 3).

### 3.2. Method Validation

The complete validation of the methods is shown in Table 3. Limit of quantification of AMPA, GLY and GLU in plasma and urine were 3, 1 and 0.3 ng/mL and 3.6, 5.1 and 0.3 ng/mL, respectively. Precision and accuracy, calculated at 10 ng/mL, were found to be <15% in all cases. Recoveries were between 45 and 84% for the analytes in plasma, with the lowest performance for GLY. In urine, recoveries were between 39 and 78%, with lower recoveries for GLY and GLU. These recoveries results were lower than other validation reports that use IS or ILIS compounds to improve the analytical parameters [27,28] but in the range of the recoveries reviewed by Wei et al. (24 to 123%) [29].

### 3.3. Study Subjects

The developed and validated methodologies were applied to plasma and urine samples obtained from subjects from the agricultural area of the province of Córdoba, Argentina. A total of 31 subjects participated in the study. Overall, 12 of them were people from the general population and 19 were occupationally exposed to pesticides. Socio-demographic characteristics of the population are informed in Table 4. All the subjects involved were men. No differences in age, weight, height, BMI, and marital status were found between groups of subjects. The only significant difference found was for the educational level, with a large number of subjects within the highest educational level in the non-occupationally exposed group. In the occupationally exposed group, all the subjects reported to have used the herbicide GLY during the week before the sampling. The non-occupationally exposed subjects declared themselves as not having used the herbicides at home.

Table 5 shows the results of the analysis of biological samples. All the analytes were detected and quantified in both matrices. Glyphosate was the most frequently detected analyte in plasma, followed by GLU and, to a lesser extent, AMPA. In urine, the most frequently detected herbicide was GLU followed by GLY and, again, the less frequently detected was the metabolite AMPA. No statistical differences between groups of subjects were found regarding the median concentration of the analytes. No correlations between the concentrations of the analytes in the different matrices were found.

## 4. Discussion

Due to the widespread use and concerns about the presence of herbicides in different matrices, several efforts were made to effectively detect the parent compound and its main metabolites, not only in environmental matrices but also in biological samples [10,30,31]. Methodologies are mainly performed for the detection of GLY and its main metabolite, AMPA, and a smaller number of studies are focused on the detection of other herbicides, such as GLU. As a result of the similar molecular characteristics of the mention herbicides, it is possible to measure them under the same chromatographic conditions [32]. However, the determination of these analytes at low concentration levels is particularly challenging due to their high polarity, water solubility, low mass, amphotericity, non-volatility property, and the lack of chromophores or fluorophores groups in their molecules. All these aspects make it impossible to determine these herbicides in the routine multi-residue analysis methods for pesticides [33]. In addition, necessary pre-treatment and derivatization steps of the samples are needed to detect the compounds [20].

Sample pre-treatment consists of a series of steps dedicated to the clean-up, concentration, and conditioning to derivatize the analytes of the sample before its detection. The derivatization step is a key issue for the detection of these compounds. Indeed, before the derivatization, conditioning steps are needed to obtain the appropriate sample for derivatization. Generally, the first step consists of strong acidification of the sample to release a stable complex that the herbicides could form with multivalent cations due to its amphoteric characteristic [17,25]. Our developed methodology involved an acidification step, for both plasma and urine samples, with HCl to reach pH = 1 (for urine samples, methodology A). We also tested other possible ways of pre-conditioning of the urine samples. Methodology B consisted of the addition of EDTA previously of the acidification step and methodology C consisted of the strong alkalinization of the urine samples with 0.6 M NaOH instead of the acidification. These two alternative pre-treatments of the sample did not improve the analytical performance of the method (Table 2) so the classical acidification of the sample was chosen. In the case of plasma samples, before the acidification step, deproteinization of the sample with ACN was needed since some molecules, like proteins, could interfere with the derivatization agents [34]. Then, a step of pH neutralization is required to obtain alkaline conditions for the derivatization step [17]. In our methods, neutralization consisted of the addition of NaOH to reach pH = 7 in both types of samples. Regarding more complex matrices, such as soil samples, urine samples needed an extra extraction step to obtain better analytical results. Our procedure consisted of the addition of a mixture of borate buffer and a potassium salt to the sample followed by shaking and sonication steps (Table 2; methodologies J and K) [10,14]. The application of this extraction step improves the recoveries of the analytes in urine samples at least twice (Table 2).

Most of the studies add IS or ILIS to the sample before the derivatization step. The use of ILIS has been widely used because it improves the method performance by minimizing variations in the composition of the samples and, so on, the matrix effect [27]. However, we could observe that the use of the ILIS increased the result of the concentration of the analytes in the biological samples, maybe due to a process of degradation of the used labeled standard.

A pre-column derivatization step was performed, as described in several analytical procedures already published, using FMOC-Cl [10,25]. The concentration of 6.5 mM FMOC-Cl derives good performance for both plasma and urine samples, without the need to increase the concentration of the derivatizer (methodology D). We also demonstrate that the dilution of the sample in the step of conditioning for the derivatization (by adjusting the volume of the sample by the addition of HPLC-grade water and borate buffer) improves the performance of the methodology considerably (methodologies E, F, G, and K). Of these methodologies, methodology G was dismissed because of the use of a special material and larger volume of the solutions and did not significantly improve the recoveries of the analytes (Table 2).

After derivatization, an off-line SPE strategy was used to clean up and concentrate the analytes. We tested two different cartridges generally used for environmental matrices, OASIS HLB and OASIS WAX [22,23,25]. The OASIS WAX cartridges did not achieve good results in terms of recoveries, so they were discarded (methodology H and I) for the analysis of urine samples. The use of the OASIS HLB cartridges showed a good performance for plasma samples and, also, showed better results (methodologies J and K) than the previously mentioned cartridge and was the selected one for the final analytical method for the urine samples. Between these two methodologies for the analysis of the real urine samples, the K one was selected because of the improvement in the recoveries of AMPA and GLU.

After having defined the analytical procedure to be used for each matrix, namely plasma and urine, several analytical parameters were evaluated to assess the analytical quality of the selected methods (Table 3). Calibration curves of the analytes responded to a linear regression (r > 0.99) over the evaluated range of concentrations. The LOQ for GLU in urine and plasma was in the same order of magnitude as that reported in the latest bibliography. The LOQs for GLY and AMPA in both matrices were an order of magnitude higher than the GLU LOQ. However, the LOQs obtained for GLY and AMPA are in the order of magnitude of the majority of the previous publications. Indeed, the developed methodology is possible for application without the need for costly automatization systems even without the use of IS or ILIS [19,27,28,29,35]. Precision and accuracy evaluated at 10 ng/mL were found to be <15% in all cases. Absolute recoveries for the analytes were between 45 and 84% in plasma and between 39 and 78% in urine. In the present study, the use of IS or ILIS was not considered due to the degradation of the used batch. Including IS or ILIS should improve the obtained recovery as is described for complex matrices [36].

There are no previous studies on the human toxicokinetic effect of GLY in plasma. Animal studies demonstrated half-life absorption after oral exposure of 2.3 h, a maximal plasma concentration of GLY approximately at 5 h after the oral dosing, and an elimination half-life of 14.4 h [37]. Also, the human toxicokinetic effect of GLY in urine was scarcely investigated [38,39]. However, the results of these two studies are very similar. They both refer to the fact that the elimination of GLY in urine is much lower in humans than in animals. The excretion half-life of GLY was between 6 and 9 h in the rapid phase and between 18 and 33 h in the slower phase [38]. Animal studies found that more than 20% of administered GLY concentrations are recovered in urine, while human studies indicated that GLY is poorly absorbed after oral doses, with urine recoveries within 1 to 6%. The low urinary excretion rates of GLY in humans is also indicative of low systemic availability; however, further research is needed on the toxicokinetic/toxicodynamic behavior of the compound. Moreover, no human study has investigated the elimination of GLY in feces, which, according to animal studies, is the main route of elimination of the unabsorbed fraction of the compound. According to Faniband et al. [38], the urine concentrations of GLY did not return to the pre-exposure values even after 80–100 h.

With regard to real plasma samples analysis, GLY was the most frequently analyte detected in plasma (32% of the total population; Table 5). Even when the toxicokinetics of GLY indicate that the herbicide is rapidly excreted in urine after being absorbed, this result shows that the population under study could be continuously exposed to the herbicide, even for general or occupationally exposed populations. In the present study, all the occupational exposed subjects reported having applied GLY during the week before the sampling, so the obtained results may be due to the recent application of the herbicide in the area under study. The majority of the previous studies only consider the measurement of GLY in plasma when it comes to poisonings [28,34,40]. In comparison with those studies, the median concentration of GLY found in the present study is at least 1000 times lower. Our result of the median concentration of GLY in the plasma of both groups of subjects is also lower than the median obtained in the general population from Thailand [41].

According to animal studies, GLU toxicokinetic is similar to GLY. A maximum plasma concentration of GLU is informed to be almost immediately after oral exposure and an elimination half-life of 4 to 5 h was reported. Rapid elimination via excreta and urine of the parent compound (approximately 70–90% and 10%, respectively) is also informed [42,43]. Glufosinate herbicide was only found in plasma samples from the general population (non-occupationally exposed subjects). Workers included in this study did not report to have recently applied GLU in the area of study and then, our results indicated that the exposure probably comes from a recent meal of the individuals [18]. This study is the first in reporting the presence of GLU in plasma samples of a population, so our findings could not be compared with any literature.

The metabolite of GLY, AMPA, was found only in one plasma sample (DF 5%) of an occupationally exposed subject (Table 5). This result could be the consequence of the direct environmental exposure to the metabolite during the working tasks due to the higher persistence of AMPA in the environment when compared to GLY.

According to the results obtained for urine samples, GLU was the most frequently analyte detected in urine (13% of the total population; Table 5). This result is in agreement with the reported toxicokinetic for the compound, which indicates its rapid elimination after being absorbed. This is also the first report on the urinary concentration of GLU in humans; consequently, we could not compare our findings with the literature.

Glyphosate was found only in urine samples of occupationally exposed subjects, indicating that their recent exposure is, maybe, due to their working conditions, for example, the re-entry to the sprayed fields. The general media (GM) obtained for GLY of this population was comparable with the GM obtained for farmers from the USA who performed the activity with less protective practices [44] and higher than the GM reported in studies carried on in an occupational exposed population from Ireland [35]. Aquavella et al. [44] reported an LOD = 1 μg/L while Connolly et al. [35] informed an LOD = 0.5 μg/L. The median concentration found in the present study was higher than that reported for a highly exposed population from Chaco (Argentina) [19] and from the general population from Germany [27]. Bressan et al. [19] reported LOQ = 0.5 μg/L and Conrad et al. [27] LOQ = 0.1 μg/L; hence, the comparison may be biased by this difference.

The metabolite AMPA was found only in one urine sample of a non-occupationally exposed subject. This aspect may reflect environmental exposure to AMPA or a differentiated capability of the individual to metabolize GLY. These findings are in accordance with toxicokinetic studies in humans, which inform us that GLY is metabolized into AMPA in less than 1% [38,39].

Comparing results obtained for each compound into a group of subjects, different patterns of body distribution could be observed. Thus, GLY was only found in plasma samples from the non-occupationally exposed group, while in the occupationally exposed subjects, GLY was present in plasma and urine samples. On the contrary, GLU was found in plasma and urine samples from the non-occupationally exposed but only in urine samples of the occupationally exposed subjects. In both cases, when the compound was found in both matrices, the DF was higher in plasma, while the concentration was much higher in urine, indicating rapid excretion of the compounds after exposure. These trends should be cautiously considered due to the small number of subjects and the low DF obtained.

Positive correlations between GLY and AMPA concentration within and between matrices are usually informed [27,45]. However, neither statistical differences between groups of subjects nor correlations between matrices were found in the present study. The lack of statistical differences and correlations between groups of subjects and matrices may be due to the number of subjects involved in the study but also because of the need for knowledge about the exposure (as time and dose).

## 5. Conclusions

The developed and validated analytical methodologies for the quantification of GLY, GLU, and AMPA were adequate to analyze the biological samples. This methodology allows carrying out the derivatization and extraction steps to be off-line, without the need to use more complex equipment. The outcomes of the performance of the analytical methodology are reliable even without the use of the ILIS. However, improved outcomes could be achieved if the ILIS is correctly used.

This study is the first report of the concentration of GLU in plasma and urine in human populations, even for occupational or non-occupational exposure to pesticides. The study is also the first to report the exposure to GLY and GLU in the population under study.

Results of the present study should be interpreted as initial information on the concentration of the herbicides in human matrices of the evaluated population because of the limitation of the study on the number of participants. Further studies should be conducted to clarify the possible health effects and to perform a risk assessment of the exposure to low levels of these herbicides in the population under study.

## Figures and Tables

**Table 1:** Instrumental analytical parameters of the studied analytes in plasma and urine.

Analyte	^a^Rt (min)	^b^Q-SRM (*m*/*z*)	^c^C-SRM (*m*/*z*)	Collision Energy (V)	^d^RF Lens (V)
AMPA	9.0	332.1–110		11	39
			332.1–136	21	39
GLY	7.6	390.1–168		16	39
			190.1–150	35	39
GLU	8.0	402.1–180		13	45
			402.1–206	20	45

^a^Rt: retention time; ^b^Q-SRM: quantification selected reaction monitoring; ^c^C-SRM: confirmation selected reaction monitoring; ^d^radio frequency lens.

**Table 2:** Recoveries (%) of the different methodologies assessed in urine.

Methodology	GLY	AMPA	GLU
A	10	22	6
B	6	21	13
C	6	19	11
D	7	22	20
E	10	29	21
F	14	56	40
G	13	56	42
H	4	19	9
I	5	36	15
J	29	71	27
K	40	78	39

**Table 3:** Method validation: limits of detection (LOD) and quantification (LOQ; µg/L), recoveries, precision and accuracy for the methodology assessed in plasma, and the selected methodology (K) for urine samples.

Analyte	LOD	LOQ	Recoveries (%)	Precision (RSD)	Accuracy (RE%)
**Plasma**					
**AMPA**	0.9	3.00	84	3	5.6
**GLY**	0.31	1.00	45	3	3.1
**GLU**	0.1	0.3	59	4	2.8
**Urine**					
**AMPA**	1.1	3.6	78	13	9.61
**GLY**	1.5	5.1	40	1	3.64
**GLU**	0.1	0.3	39	3	3.05

**Table 4:** Socio-demographic characteristics of subjects occupationally (*n* = 19) and non-occupationally (*n* = 12) exposed to pesticides from the Province of Córdoba.

	Non-Occupationally Exposed	Occupationally Exposed	*p*-Value
	Mean ± SD or %	Mean ± SD or %	
Gender	Male	Male	
Age ^a^	45 ± 10	43 ± 10	0.4858
Height ^a^	176.83 ± 9.89	172.26 ± 7.89	0.2552
Weight ^a^	85.83 ± 16.10	89.86 ± 12.55	0.5271
BMI ^b,c^			0.621
Normal	17	5	
Overweight	50	47	
Obesity	33	47	
Education level ^c,d^			**0.022**
Elementary	0	16	
Middle	10	32	
High school	10	32	
University	80	21	
Marital status ^c,e^			0.408
Married	64	68	
Divorced or separated	9	0	
Widower	0	0	
Single	27	32	
GLY application during the previous week	0	100	
GLU application during the previous week	0	0	

^a^ *p* value of the comparisons of continuing variables between non-occupationally and occupationally exposed groups was calculated using *t*-test. ^b^ BMI (body mass index, kg/m^2^). ^c^ Association between exposure and categorical variables was calculated using a chi-squared test. ^d^ Educational level was categorized as either elementary (1), middle (2), high school (3), or university (4). ^e^ Marital status was categorized as: Married (1), Divorced or separated (2), Widower (3), Single (4).

**Table 5:** Results of the analysis of real samples (ng/mL) expressed as detection frequencies (DF), geometric means (GM), confidence interval (CI), median, range (min–max), and 95th percentile (p95) in subjects occupationally (*n* = 19) and non-occupationally (*n* = 12) exposed to pesticides from the Córdoba province.

Analyte	Matrix	Non-Occupationally Exposed						Occupationally Exposed						*p*-Value ^a^
		DF (%)	GM	CI	Median	Range	p95	DF (%)	GM	CI	Median	Range	p95	
**AMPA**	**Plasma**	0	<LOD	-	<LOD	-	-	5	6.800	-	6.80	-	-	-
	**Urine**	8	1.700	-	1.70	-	-	0	<LOD	-	<LOD	-	-	-
**GLY**	**Plasma**	50	4.378	2.228–8.603	3.75	2.40–14.10	5.40	21	4.456	1.361–14.584	5.70	1.50–8.10	5.90	0.5224
	**Urine**	0	<LOD	-	<LOD	-	-	11	10.205	0.634–164.374	10.45	8.20–12.70	8.20	-
**GLU**	**Plasma**	25	0.978	0.521–1.835	0.90	0.80–1.30	0.90	0	<LOD	-	<LOD	-	-	-
	**Urine**	17	9.592	5.647–16.291	9.60	9.20–10.00	9.20	11	11.739	0.006–21,452.140	13.85	6.50–21.20	6.50	1.0000

^a^ Mann–Whitney U tests for comparisons between non-occupationally and occupationally exposed groups. Only results > LOQ were used.

## Data Availability

Data are unavailable due to privacy or ethical restrictions.

## Supplementary Materials

The following supporting information can be downloaded at https://www.mdpi.com/article/10.3390/toxics11121020/s1. Table S1: Summary of the methodology used for GLY, AMPA, and GLU analysis in plasma; Table S2: Summary of the methodology used for GLY, AMPA, and GLU analysis in urine.
